# Patient-Reported Outcome After Extended Total Mesorectal Excision for Locally Advanced Rectal Cancer in Male Patients

**DOI:** 10.1007/s13193-025-02237-4

**Published:** 2025-04-25

**Authors:** Akash Mor, Geet Midha, Tejas Vispute, Ankit Sharma, Mufaddal Kazi, Ashwin Desouza, Avanish Saklani

**Affiliations:** 1 Department of Surgical Oncology, Tata Memorial Hospital and Homi Bhabha National Institute, Mumbai, India; 2 Division of Colorectal Surgery, Department of Surgical Oncology, Advanced Centre for the Treatment, Research, and Education in Cancer, Tata Memorial Hospital and Homi Bhabha National Institute, Mumbai, India; 3 Division of Colorectal Surgery, Department of Surgical Oncology, Tata Memorial Hospital and Homi Bhabha National Institute, Dr Ernest Borges, Marg, Parel, Mumbai, 400012 Maharashtra India

**Keywords:** Extended total mesorectal excision (eTME), Locally advanced rectal cancer (LARC), Circumferential resection margin (CRM), International prostatic symptom score (IPSS), International Index of erectile function score (IIEF), Quality of life (QOL)

## Abstract

This study looks at sexual and urinary dysfunction and quality of life in male patients undergoing extended total mesorectal excision. This cross-sectional study used International Prostatic Symptom Score (IPSS) and the International Index of Erectile Function Score (IIEF) questionnaire-based retrospective analysis of male patients who underwent extended total mesorectal excision from 2015 to 2022. Quality of life was assessed using EORTC QLQ C-30 and EORTC QLQ CR-29. Sixty-eight male patients were included, with a median age of 44 years. Urinary retention and incontinence occurred in 10.3% of patients, and 2 required lifelong catheterization. Nineteen percent and 49% patients had severe urinary and sexual dysfunction as per IPSS and IIEF scores. As per the EORTC C-30 QOL analysis, participants scored a global health status mean score of 33.3 with a standard deviation of 10.76. The highest functional scale score was for cognitive functioning: 78.7 ± 18.67. The symptom scale ranged from 9.30 ± 13.26 for nausea and vomiting to 44.19 ± 27.9 for financial difficulties. According to the EORTC CR 29, impotence (43.41 ± 55.17) and problems with stoma care (37.20 ± 22) scored highest. On the function scale, anxiety about future health (62.79 ± 24.35), interest in sex (65.11 ± 45.4), and body image (65.12 ± 16) scored lowest in this order. The patient had significant urinary and sexual symptoms, resulting in concern about weight, loss of interest in sex, and anxiety about future health. In a high-volume , eTME is not without urinary and sexual dysfunction.

## Introduction

Forty percent of Indian patients present with locally advanced rectal cancer(LARC), which requires resection beyond the mesorectal plane despite neoadjuvant therapy [1]. Patients with tumors involving the mesorectal fascia need surgical resection beyond the conventional total mesorectal plane; many of them will need exenteration [2, 3]. The prognostic significance of achieving negative CRM in reducing local and distant failures is proven in the literature [2]. However, recent trends have focused on improving our ability to risk-stratify patients and tailoring treatment to achieve the best oncologic outcome while limiting the impact on long-term quality of life. Exenterative surgeries have been standard of care for tumor beyond mesorectal fascia [2]. These exenterative surgeries are associated with significant perioperative morbidity and affect quality of life [5, 6].

Extended total mesorectal excision(eTME) is defined as a partial resection/shave of adjacent organ(s) of the rectum, such as the posterior wall of the prostate or the vagina, the uterus, the seminal vesicles, the hypogastric plexuses, the ureter and the bladder, en bloc with TME with curative intent, to achieve an R0 resection which often involves resection of sympathetic or parasympathetic supply ( Unilateral or Bilateral) [3, 4]. This approach has provided an R0 resection rate of 90.6% with a local recurrence of 7.3% at a median follow-up of 28 months [4]. Hence, eTME is a safe option for patients with involved mesorectal fascia (clinically T3) with an acceptable R0 resection rate and survival outcomes [4]. We have previously reported physical and psychological changes affecting urinary and sexual health in female patients undergoing multi-visceral resection [5].

There is very little data on functional outcomes after extended TME. Do these patients live on a long-term catheter or lifelong clean intermittent catheterisation and do some patients recover their sexual function, is the question. This snapshot focuses on short and intermediate sexual and urinary dysfunction, global quality of life, and colorectal cancer specific quality of life in male patients undergoing e TME.

## Method

### Study Design

A retrospective analysis of a prospectively maintained database of all male patients undergoing eTME for LARC is performed.

### Inclusion Criteria

All male patient undergoing extended total mesorectal excision included.

### Exclusion Criteria

All exenterations and histologies other than adenocarcinoma were excluded. All patients who are lost to follow up, expired, or had recurrence were excluded.

### Staging and Neoadjuvant Treatment

Staging included digital per rectal examination, colonoscopy with biopsy, CTchest, and abdomen and MRI pelvis for local staging. After the above investigation, all patients were discussed in multidisciplinary meeting. Neoadjuvant therapy (radiation schedule and chemotherapy) individualized as per MDT decision. After 6 weeks, a response assessment with MRI, including a specific diffusion-weighted sequence, was done. Fixity to adjacent organs was confirmed with a digital rectal examination. Based on the clinic-radiological findings, a further surgical plan of eTME vs. exenteration was made.

### Surgery

Patients underwent eTME using open or minimally invasive approach. Over the later years with increasing experience MIS was the preferred approach for eTME [6]. Techniques of extended TME have been published by us before [7–9].

Following quadrant-based classification of eTME has been proposed by our institute which was already described in previous paper [4]-

1. Anterior quadrant includes resection of posterior vaginal wall, prostatic shave, bladder peritonectomy, seminal vesicle excision, or partial cystectomy.
2. Posterior quadrant involves resection of presacral fascia.
3. Lateral quadrant involves vascular approach to remove all fibrofatty tissue around the vessel as a margin, hypogastric nerves, lateral muscles such as pyriformis or obturator internus or distal branches of iliac vessels.
4. Multi-quadrant resection may be required in some patients to achieve circumferential resection margin.
5. Lateral pelvic lymph node dissection performed in patients with radiologically significant nodes post neoadjuvant treatment.

### Postoperative Measures and Follow-Up

The urinary catheter was kept for 3–14 days in patients undergoing eTME. Patients going in to retention were recatheterised and subsequently taught clean intermittent catheterization (CIC), if they failed trial without catheter at 4 weeks. At 4 weeks, patients had ultrasound of bladder to calculate post void residue. Post void residue more than 50 ml or symptomatic were recatheterised and subsequently taught CIC. This was also done for patients who were catheter free but complained of urinary dysfunction subsequently. All patients, after completion of treatment, were kept under standard follow-up as per NCCN recommendations 3 monthly for 2 years, 6 monthly for the next 3 years; annually after that, Sr. CEA and clinical examination at every visit; colonoscopy at 1, 3, and 5 years; and CECT every year for 5 years.

### Variables Collected

The dataset includes demography, neoadjuvant treatment received, type of surgery and quadrant removed, intraoperative complications, postoperative day of urinary catheter removal, any post-urinary catheter removal complications such as urinary retention or incontinence, and the requirement of re-catheterization or clean intermittent self-catheterization.

### Outcome Measure

Patients were contacted telephonically, or physically during routine follow-up and questions were asked according to the International Prostatic Symptom Score (IPSS) and the International Index of Erectile Function Score (IIEF), and EORTC C-30 and CR-29, and scores were calculated and stratified. The median time to questionnaire was 30 months.

Using the variables in the International Prostatic Symptom Score (IPSS) and the International Index of Erectile Function Score (IIEF), the final score is calculated. The IPSS score is divided into three categories: mildly symptomatic for a score of 0–7, moderately symptomatic for a score of 8–19, and severely symptomatic for a score of 20–35 [8]. Also, the final IEF score is divided into no erectile dysfunction for a score of 22–25, mild erectile dysfunction for a score of 17–21, mild to moderate erectile dysfunction for a score of 12–16, moderate erectile dysfunction for a score of 8–11, and severe erectile dysfunction for a score of 5–7 [9]. The correlation of IPSS and IIEF to the type of neoadjuvant therapy, type of surgery, and quadrant resected was evaluated.

Single time assessment of quality of life was undertaken for the patient who consented for the study. EORTC C-30 and CR-29 questionnaire were used in the patient preferred language [10, 11]. Patients were provided with the questionnaire either in-person during the follow-up visits or telephonically when a physical visit was not possible. Consent was obtained in both situations. For patients that were unable to read and understand or if questionnaire is not available in preferred language, the questionnaire was administered by clinician. patients. The raw score was transformed into linear score of 0–100 using EORTC guidelines. The missing items were dealt as per EORTC guidelines. A high score on functioning scales represents a better level of functioning whereas a high score on symptom scales represents worse or severe symptoms.

### Statistical Analysis

Data were recorded on SPSS and analyzed using SPSS version 23. Continuous variables were recorded using the mean, and standard deviation. All continuous variables were compared using the Student *t*-test. The value of *p* ≤ 0.05 was considered significant. Comparative analysis was conducted by using the Pearson *χ*^2^ statistic or Fisher’s exact test for categorical data and the paired *t*-test and Wilcoxon signed rank test for the investigation of sequential continuous variables. Microsoft Word and Excel have been used to create graphs, tables. The EORTC C-30 and CR-29 transformed scores described using mean and standard deviation (SD).

## Results

### Baseline Characteristics

**Fig 1:**
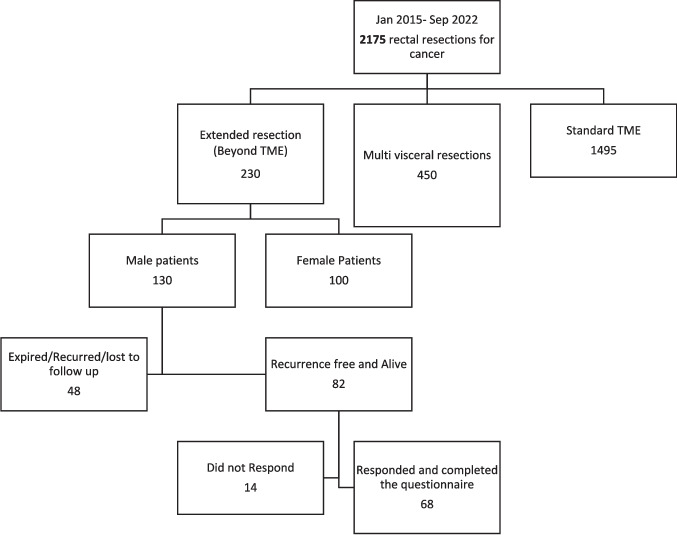
Patient selection

Sixty-eight male patients were included (Figure 1), with a median age of 44 years at the time of surgery. The majority had (35.51%) low rectal tumors with 56% (38 patients) receiving neoadjuvant long-course chemo-radiation and 71% (48 patients) undergoing laparoscopic surgery. Fifty-two percent (36 patients) underwent abdominoperineal resection, while the sphincter was preserved in 6% (3 patients) by inter-sphincteric dissection and in 42% (29 patients) by low anterior resection. Most commonly extended resection was performed in anterior quadrant (41,60%). Clavien-Dindo, grade IIIa complications were discovered in 21% (14 patients), with perineal wound breakdown being most frequent. Hypogastric nerves were sacrificed unilaterally in 45.58% (31 patients) and bilaterally in 7.4% (5 patients) (Table 1).

**Table 1:** Baseline characteristics

Demographic	Value
No of patients	68
Median age	44.2 +/− 14.9 years
T stage	T3-27 (39%) T4-41 (61%)
N stage	N0-12 (17.6%) N1-25 (36.8%) N2-31 (45.6%)
Location	Rectosigmoid 8 (12%) Upper rectum (12–15 cm)−12(18%) Mid-rectum (7–11 cm)−13 (19%) Lower Rectum (0–6 cm) 35 (51%)
Median distance from Anal verge (in cm) (IQR)	4 cm (2 cm; 7 cm)
Neoadjuvant treatment	Long course CTRT-38 (56%) Short course RT f/b Consolidation chemo-23 (34%) Upfront surgery-7 (10%)
Approach of surgery	Laparoscopic – 48(71%) Robotic-3 (4%) Open-17 (25%)
Type of surgery	Abdomino-perineal resection-36 (52%) Intersphincteric resection – 3 (6%) Low anterior resection-29 (42%)
eTME quadrant	Anterior −41 (60%) Lateral- 14(21%) Posterior- 11 (16%) Multi-quadrant- 2 (3%)
Lateral pelvic lymph node dissection	10 (17%)
Hypogastric nerve sacrificed	Unilateral −31 (45.58%) Bilateral – 5 (7.4%)
Clavien-Dindo grade IIIa complication	14 (21%)
Median catheter removal day (IQR)	7 (4;14)
Post catheter removal complication requiring catheter insertion	Retention-7 (10.3%) Incontinence- 7 (10.3%)
Permanent clean intermittent self-catheterization	2(3%)

The median urinary catheter removal day was 7 days (range: 3–60 days). 21% (14 patients) required catheterization, 10.3% (7 patients) due to retention, and 10.3% (7 patients) due to incontinence. Two patients required clean, intermittent self-catheterization. The median time to questionnaire was 30 months.

### International Prostatic Symptom Score (IPSS)

**Fig 2:**
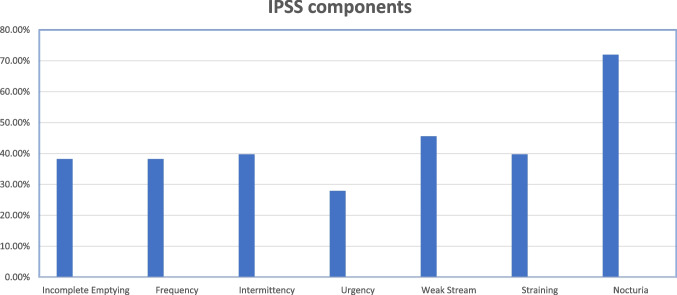
IPSS components and percentage of patient with the same

**Fig 3:**
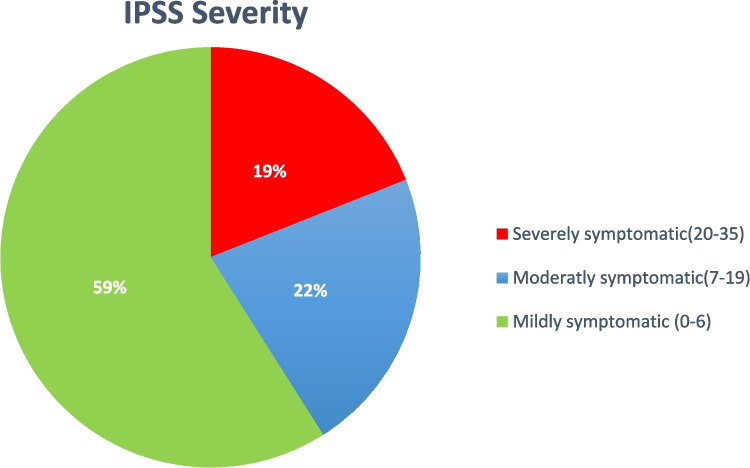
IPSS severity

After evaluating the IPSS questionnaire, 59% (40 patients) were mildly symptomatic (IPSS score of 0–7), 22% (15 patients) were moderately symptomatic (IPSS score of 8–19), and 19% (13 patients) were severely symptomatic (20–35)(Figure 2). The IPSS component showed that nocturia (72% of patients) was the commonest symptom, followed by weak stream (46%), intermittency and straining (40%), incomplete emptying and frequency (38%), and urgency (28%) (Figure 3). Forty percent of patients are taking medication for lower urinary tract symptoms (LUTS) at a median follow-up of 30 months. There was no difference in IPSS severity with sphincter-preserving vs. abdomino-perineal resection (*p*−0.275). There is no difference between patients receiving neoadjuvant long-course vs. short-course radiation therapy (*p*−0.4). Severe symptoms are commonly associated with anterior quadrant resection (84%) eTME followed by lateral quadrant (16%).

### International Index of Erectile Function Score (IIEF)

**Fig 4:**
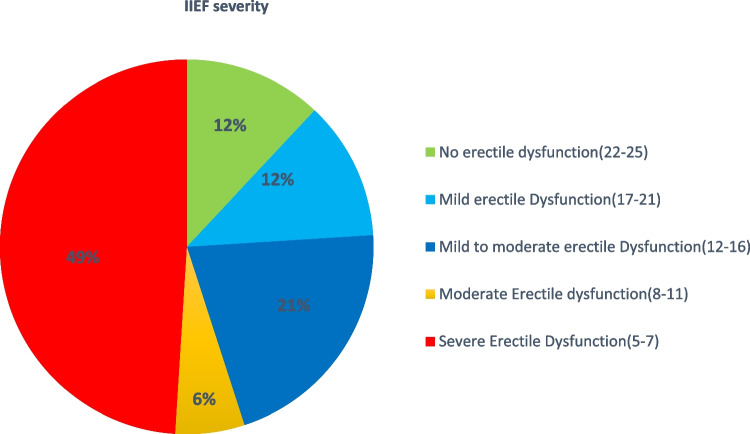
IIEF severity

After IIEF evaluation in 51 patients, 49% (25 patients) had severe erectile dysfunction, and 6% (3 patients) had moderate erectile dysfunction. While 12% (6 patients) had no erectile dysfunction, 21% (11 patients) had mild to moderate erectile dysfunction, and 12% (6 patients) had mild erectile dysfunction (Figure 4). There is no difference in IIEF severity about sphincter-preserving surgery or abdominoperineal resection (*p*−0.487). There is no difference concerning the type of neoadjuvant treatment (*p* < 0.45). Severe erectile dysfunction was more prevalent in the anterior quadrant of eTME (60%), followed by the lateral quadrant (36%). Twenty-five percent of patients were sexually inactive at the time of analysis irrespective of erectile dysfunction. Only 9 patients had antegrade ejaculation out of 25 patients who answered the question.

### Quality of Life Analysis (QOL)

Out of 68 patients, 43 were able to fill out the questionnaire. As per the EORTC C 30 QOL analysis, participants scored a global health status/QOL mean score of 33.3 with a standard deviation of 10.76. Functional scale scores ranged from 78.7 ± 18.67 for cognitive functioning to 61.62 ± 14.7 for emotional functioning. Symptom scales ranged from 9.30 ± 13.26 for nausea and vomiting to 44.19 ± 27.9 for financial difficulties (Table 2).

**Table 2:** Transformed scale scores: EORTC QLQ C-30

	Mean ± SD	Range
*Global health status /QOL*	33.3 ± 10.76	(33.3–83.3)
*Function scale*		
Physical functioning	77.2 ± 13.82	(40–100)
Role functioning	72 ± 17.3	(33.3–100)
Emotional functioning	61.62 ± 14.7	(33.34–91.67)
Cognitive functioning	78.7 ± 18.67	(33.34–100)
Social functioning	65.11 ± 16.9	(16.67–100)
*Symptom scale*		
Fatigue	28.16 ± 14.8	(0–66.67)
Nausea and vomiting	9.30 ± 13.26	(0–66.67)
Pain	21.70 ± 16.48	(0–66.67)
Dyspnoea	27.90 ± 26.15	(0–66.7)
Insomnia	35.66 ± 22.30	(0–66.67)
Loss of appetite	35.66 ± 22.30	(0–66.67)
Constipation	10 ± 20	(0–66.67)
Diarrhoea	18.6 ± 25	(0–100)
Financial difficulty	44.19 ± 27.9	(0–100)

According to the EORTC CR 29, impotence 43.41± 55.17, problems with stoma care 37.20 ± 22, urinary frequency 33.34 ± 21.5, and anorectal pain 30.23 ± 26 scored highest in this order. On the function scale, anxiety about future health 62.79 ± 24.35, interest in sex 65.11 ± 45.4, and body image 65.12 ± 16 scored lowest in this order (Table 3).

**Table 3:** Transformed scale scores: EORTC QLQ CR 29

	Mean ± SD	Range
*Symptom scale*		
Urinary frequency	33.34 ± 21.5	(0–100)
Urinary incontinence	19.3 ± 25.4	(0–100)
Dysuria	11.6 ± 19.9	(0–66.7)
Abdominal pain	10 ± 17.11	(0–66.7)
Anorectal/gluteal pain	30.23 ± 26	(0–100)
Bloating	25.58 ± 17.6	(0–66.7)
Blood or mucus in stools	3.1 ± 7.5	(0–33.4)
Dry mouth	5.4 ± 17.71	(0–66.7)
Hair loss	17.8 ± 27.55	(0–100)
Loss of taste	5.42 ± 14.42	(0–66.7)
Bowel frequency	22.48 ± 19.20	(0–100)
Problems with stoma care(N-29)	37.20 ± 22	(0–66.7)
Impotence	43.41 ± 55.17	(0–100)
*Function scale*		
Anxiety about future health	62.79 ± 24.35	(33.4–100)
Concerns with weight	72.86 ± 24.4	(0–100)
Body image	65.12 ± 16	(33–100)
Interest in sex	65.11 ± 45.4	(0–100)

### Questionnaire Non-responders and Excluded Patients

Clinical, surgical, and postoperative characteristics of patients who did not respond to the questionnaire are outlined in Table 4. There was no significant difference in these patients compared to the included patients in terms of age, tumor location, clinical T and N stages, neoadjuvant treatment, type of surgery and approach to surgery, extended TME quadrant, and post-operative urinary catheter removal day.

**Table 4:** Baseline characteristics of questionnaire non-responders and responders

Demographic	Questionnaire responders	Questionnaire non-responders
No of patients	68	14
Median age	45.2 +/− 12.8 years	43 +/− 14.2 years
*T* stage	T3-27 (39%) T4-41 (61%)	T3-4 (28.6%) T4-10 (71.4%)
*N* stage	N0-12 (17.6%) N1-25 (36.8%) N2-31 (45.6%)	N0-1 (7.1%) N1-4 (28.6%) N2-9 (64.3%)
Location	Rectosigmoid 8 (12%) Upper rectum (12–15 cm) 12 (18%) Mid-rectum (7–11 cm) 13 (19%) Lower rectum (0–6 cm) 35 (51%)	Rectosigmoid 3 (21.4%) Mid-rectum (7–11 cm) 7 (50%) Lower rectum (0–6 cm) 4 (28.6%)
Median distance from anal verge (in cm) (IQR)	4 cm (2 cm; 7 cm)	4.1 cm (2 cm; 7 cm)
Neoadjuvant treatment	Long course CTRT- 38 (56%) Short course RT f/b consolidation chemo-23 (34%) Upfront surgery-7 (10%)	Long course CTRT- 6 (43%) Short course RT f/b Consolidation chemo-5 (36%) Upfront surgery-3(21%)
Approach of surgery	Laparoscopic – 48(71%) Robotic- 3 (4%) Open- 17 (25%)	Laparoscopic – 12(86%) Robotic- Nil Open- 2 (14%)
Type of surgery	Abdomino-perineal resection-36 (52%) Intersphincteric resection – 3 (6%) Low anterior resection- 29 (42%)	Abdomino-perineal resection-3 (21%) Intersphincteric resection – 3 (21%) Low anterior resection- 8 (58%)
eTME quadrant	Anterior −41 (60%) Lateral- 14(21%) Posterior- 11 (16%) Multi-quadrant- 2 (3%)	Anterior −11 (79%) Lateral- 2(14%) Posterior- 1 (7%)
Lateral pelvic lymph node dissection	10 (17%)	1 (7%)
Hypogastric nerve sacrificed	Unilateral −31 (45.58%) Bilateral – 5 (7.4%)	Unilateral −5 (45%) Bilateral – 2 (14%)
Clavien-Dindo grade IIIa complication	14 (21%)	2(14%)
Mean catheter removal day	10 days (range 3–60) 7 (4;14)	8 days (range 3–18) 8(5:16)
Post catheter removal complication requiring catheter insertion	Retention-7 (10.3%) Incontinence- 7 (10.3%)	Retention-1 (7%)
Permanent clean intermittent self-catheterization	2(3%)	Nil

## Discussion

In this study, we report urinary and sexual dysfunction in patients undergoing eTME. We reported that 20% of patients had moderate to severe urinary dysfunction. Forty-nine percent had severe sexual dysfunction at 30 months, which was resolved in most of the patients undergoing TME [12]. This can be correlated with neuroanatomy as eTME involves one plane beyond the TME plane, which leads to permanent neuronal damage whereas TME leads to neuropraxia which get resolved over a period of time [12]. These symptoms were severe in patients undergoing anterior quadrant and lateral quadrant eTME. Forty percent of patient taking medication for lower urinary tract symptom at 30 months. Two patient required clean intermittent self-catheterization.

There is limited literature available to review these functional outcome in patients undergoing extended Total mesorectal excision [13]. One series reported eight patients undergoing open TME with seminal vesicle excision for three recurrent and five upfront case. It reported two patient with severe urinary dysfunction and five patient with severe erectile dysfunction .It did not use any pre-validated questionnaire [13]. There is immense literature on significant effect of pelvic exenteration of quality of life is available [14, 15]. The data on outcomes of patient undergoing eTME is limited.

A single institution study presented the large series of patients undergoing extended TME and their survival outcomes. eTME has the R1 resection rate was 10.4%, the local recurrence rate was 7.3%, and 3-year DFS and OS rates were 66.7% and 80.4%, respectively. These survival outcomes were compared with patient undergoing exenterative surgery and safety and feasibility of eTME has been proven [4]. But functional outcomes are yet to be compared. A study focusing on anterior quadrant eTME has shown an acceptable R0 resection rate with urethral damage in 36% of patients, resulting in urinary fistulas in 18% of patients [16].

QOL in patients undergoing TME as per EORTC C-30 has a global health status score of 61.15 ± 23.35, whereas in eTME, it resulted in 33.3 ± 10.76. Cognitive functioning, followed by physical functioning, scored highest, which is comparable with patients undergoing eTME [17]. Fatigue scored highest, and nausea and vomiting scored lowest on the symptom scale in patients undergoing TME. The overall function score was comparable in patients undergoing TME and eTME. Functional scores on the EORTC CR-29 were highest for weight, body image, and anxiety, which is comparable with the current data. Urinary and sexual symptom scores were higher in patients undergoing eTME compared to TME [17–19].

Our center evaluated functional outcome in female patient undergoing multi visceral resection. Amongst the symptom scales, patients reported the most troubles with urinary frequency (mean, 69.6; SD, 9.9), pain during intercourse (mean, 44; SD, 40.7), and bowel frequency (mean, 36.9; SD,10.7) in this order. Amongst the functional scales, anxiety about future health (mean, 42.5; SD, −018.9) and interest in sex (mean, 57.2; SD, 33.2) scored the lowest, suggesting poor functioning in these aspects of life [20].

Although this study has small sample size, it gives snapshot of effect of eTME on functional and quality of life. These symptoms can be managed with pre-operative counselling and evaluation. Also, early management of these symptoms with early referral to urology and andrology clinic, social and psychological support will help in improving the quality of life.

## Limitations

This is a single-center retrospective study with a small sample size. Many patient data points are missing due to death, recurrence, failure to follow up, or not answering the call. Evaluation is completely based on a telephonic patient-based questionnaire. A pre-operative objective evaluation of urinary function and post-operative flowmetry and evaluation of the cause were not available. No historical controls were available to make comparisons. The influence of treatment for lower urinary symptoms on scores was not available. Cultural differences regarding hesitancy to answer questions and underreporting of symptoms may underestimate the true burden of dysfunction. Factor predicting dysfunction could not be assessed due to the small sample size. Further prospective studies, proper pre-op evaluation using uroflowmetry, and post-op follow-up are necessary. Predictors of IPSS and IIEF severity except the anterior and lateral quadrants were not shown.

## Future

A prospective study with a preoperative evaluation of urinary and sexual dysfunction and a postoperative longitudinal analysis of function with interval uroflowmetry must be conducted. This will give us the true burden of urinary and sexual dysfunction. This will help in proper preoperative counseling and postoperative treatment. This will help in early referral to urology and andrology for symptom management.

## Conclusion

This study demonstrates the approximate burden of urinary and sexual dysfunction in male patients undergoing extended total mesorectal excisions for locally advanced rectal. eTME significantly affects emotional, cognitive, psychosocial function, resulting in significant financial difficulty. The patient had significant urinary and sexual symptoms, resulting in concern about weight, loss of interest in sex, and anxiety about future health. In a high-volume center with experienced hands, eTME is not without sexual and urinary dysfunction. However, no patient required permanent catheterization. A survivorship clinic to look at patients undergoing eTME, or exenteration, is the need of the day. This may avoid long-term renal damage due to urinary dysfunction. The role of drugs or prostheses to improve erectile dysfunction in such patients needs further evaluation. Accordingly, pre-operative counseling and evaluation help in safe post-operative management and follow-up.
